# Detecting subsurface diseases on airport road surface based on an improved SSD algorithm

**DOI:** 10.1371/journal.pone.0327522

**Published:** 2025-07-23

**Authors:** Mengmeng Pan, Huiguang Chen, Lipeng Yang, XianRong Jiang

**Affiliations:** 1 Cloud Network Operating System Research and Development Center, China Telecommunications Corporation, Beijing, China; 2 Cloud Network Operating System Research and Development Center, China Telecommunications Corporation, Guangzhou, China; China University of Mining and Technology, CHINA

## Abstract

Due to the frequent impact of aircraft takeoff and landing and the influence of weather temperature changes, airport roads will have different types of underground diseases (DT-CRACK, DT-GAP, DT-LACUNAS and DT-SUBSIDENCE), which affect the road performance and service life, cause safety accidents, and result in a great loss of manpower and material resources. Facing the radar data of underground hidden diseases of airport roads with low recognition and high noise intensity, it is inefficient to recognize the diseases by manual identification, and it is difficult to achieve accurate differentiation and localization of the diseases by the existing detection methods. We analyze and propose an improved algorithm EFA-SSD (Enhanced Feature Aggregation SSD) for automatic detection of airport road subsurface diseases, which solves the problems of strong noise background conditions, severe interference of morphological features of different types of subsurface diseases, and low target recognition. Our model designs RFB module with wider receptive field in the network layer, which effectively suppresses the noise interference around the disease and extracts more disease features from the original radar data; in addition, the detailed texture features of different types of diseases are captured by fusing the shallow features of the model network, which realizes the classification and localization of different types of diseases; and the attention mechanism of spatial channel is introduced to enhance the feature expression ability and improve the generalization ability of the model. The spatial channel attention mechanism is introduced to enhance the feature expression and generalization ability of the model. Compared with the existing classical target detection algorithms, EFA-SSD has the highest mean average precision (mAP) in detecting four types of subsurface diseases, which provides a new idea for subsurface disease detection and contributes to the protection of aviation safety.

## Introduction

**Fig 1:**
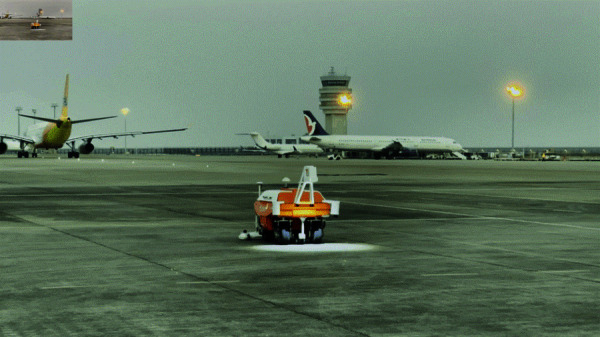
Robot performing airport road surface inspection operations.

With the rapid growth of aviation business, the underground structure of the airport road surface under the repeated impact loads of aircraft, the phenomenon of broken plate, collapse, etc., a serious threat to the safety of the road surface make and cause the loss of human and material resources. Therefore, structural defect detection has become an important task to monitor the health of airport roads, especially the early detection of defects such as underground cracks, dehollowing, spalling, and settlement. However, traditional manual inspection methods are time-consuming, laborious and error-prone, and cannot meet the needs of rapid development. Unlike highway, railroad, and bridge roadway structures, the underground structure of airport roadways consists of multiple layers, and diseases inevitably appear in these layers due to precipitation, temperature difference, and loading. Ground-penetrating radar [24,30] has become an important inspection tool suitable for uninterrupted airport operation due to its fast detection speed, high efficiency and low impact on airport operation. Fig 1 shows the schematic diagram of ground-penetrating radar working on the airport roadway surface.

**Fig 2:**
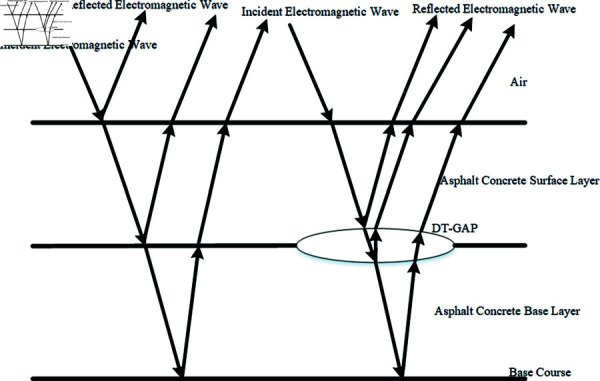
Working principle of ground-penetrating radar for disease detection.

Due to the five-layer fixed laminar structure of the airport road and the difference in the dielectric constant of the non-magnetic medium, a good electromagnetic wave reflecting interface is formed, which enables the ground-penetrating radar to effectively reflect the electromagnetic wave when detecting the underground disease [25]. When encountering road underground structural layer breakage (e.g., Fig 2 shows ground-penetrating radar detecting deconstruction disease), the radar detection results will show obvious characteristic reflections. Ground-penetrating radar is used for data acquisition at airports by time-triggered movement along the horizontal direction at equal intervals to collect data [11].

**Fig 3:**
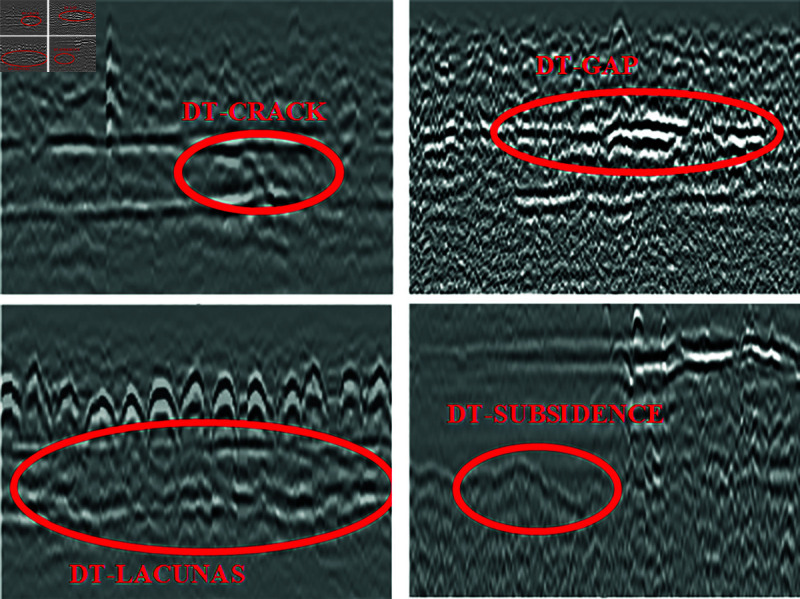
Radar diagram of various types of diseases.

These include DT-CRACK [12] (caused by shrinkage of cement materials and uneven construction), DT-GAP [13] (occurring between layers, which affects road loading capacity and may lead to fracture), DT-LACUNAS [14] (disease caused by localized porosity in the sub-structural layer of an airport’s road, and later deterioration leading to roadway caving in and settlement), and DT-SUBSIDENCE [15] (caused by insufficient soil compaction and environmental factors), which are hidden diseases that may cause safety incidents when aircraft pass by. Fig 3 shows the radar diagram for various types of diseases.

## Related work

The following three methods are currently available for the detection of subsurface disease on airport pavements:

(1) Methods of signal processing

Signal processing-based methods usually require a large amount of preprocessing and computation, mainly curve fitting method, S-transform method, wavelet transform method, phase analysis method and so on. Park *et al*. [1] effectively extracted and processed underground object signal features by phase analysis of ground-penetrating radar data, but the method is very sensitive to noise and difficult to adapt to complex environments; Al-Qadi *et al*. [2] used the wavelet transform method to analyze the radar waveform graphs, but due to the complexity of the radar waveform features, the method extracted the feature representation is limited and it is more difficult to obtain representative features.

(2) Machine learning methods

By manually designing the target features and then combining them with traditional machine learning methods [29] to detect sub-surface diseases on airport roadway surfaces, the main methods are support vector machine methods [27], scale invariant feature transform (SIFT) algorithms, and Hough’s transform method, etc. Karem *et al*. [3] used ground-penetrating radar to detect sub-surface diseases on roadway bases, and by invoking the scale invariant feature transform algorithm on the radar map was obtained for feature extraction; however, the roadbed lesions were of various morphologies, and the lesion features obtained were of a single morphology, with limited ability of artificial feature representation; Windsor *et al*. [4] used gray scale histogram and Hough transform method to artificially design the lesion features, but it could not provide scientific basis for the detection of lesions in the face of strong noise conditions.

(3) Deep learning methods

Deep learning [28,31,32] based methods are now also widely used in the field of target detection, which also includes in the task of underground disease target detection. Ding Z *et al*. [5] applied CNN to extract meaningful features from radar, but this kind of algorithm is difficult to realize the accurate localization of targets; Fang Z *et al*. [6] )used Faster R-CNN to identify hyperbolic features in gray-scale images, which can achieve the classification and localization of disease targets in radar maps, but did not take into account the complex radar data distribution characteristics of diseases, and was ineffective in detecting different types of diseases. The lightweight YOLO [7,26] series of algorithms meets the needs of real-time detection, but performs poorly in the face of complex scenarios; RetinaNet [8], RFBNet [9], and SSD [10] algorithms are unable to meet different disease morphology features due to the lack of complementarity between feature layers when performing underground disease detection in airports, which cannot meet the diversity of different disease morphology feature changes, especially in the face of complex waveform radar data detection is not effective.

The structural layer of airport road surface has non-uniformity and strong attenuation, and the electromagnetic wave propagation environment of ground-penetrating radar is complicated and variable, resulting in the radar data characteristics of underground diseases being seriously interfered. Artificial disease identification is inefficient and subjective, and cannot provide scientific basis for large-scale road maintenance and repair in time; moreover, the road structure has various forms of hidden diseases, and it is difficult to meet the needs of practical application in terms of identification accuracy. In the face of complex radar data, both manual data analysis and traditional machine learning methods have the disadvantages of one-sided information, high cost, time-consuming and laborious, and are unable to flexibly differentiate the changing appearance of various types of diseases.

## Methods

Various causative factors affect the generation and development of hidden diseases on airport roads, and the disease generation mechanism is ambiguous, random and dynamic; directly inputting radar data images into a convolutional neural network can reduce a large amount of preprocessing, and the convolutional neural network can automatically learn the disease features and be used for the detection after many iterations [23,33]. Therefore, the use of deep neural network models to detect airport road subsurface disease radar data can effectively reduce the complex preprocessing work.

### Structure of EFA-SSD

**Fig 4:**
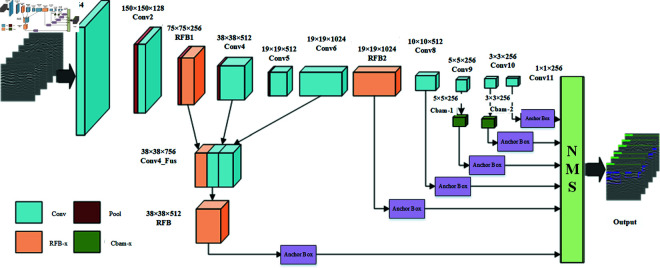
Structure of the EFA-SSD network model.

The SSD network optimizes the detection results by setting different proportions of prior frames on six different sizes of feature maps. However, the SSD network only relies on the conv4_3 layer to extract small target features, which lacks semantic information and has a limited number of convolutional kernels, resulting in insufficient accuracy for small target detection. In addition, the independent extraction of different scale feature layers does not take into account the complex radar data waveforms of subsurface diseases, which makes it difficult to accurately distinguish them in low discrimination and strong noise environments. For this reason, an improved EFA-SSD algorithm is proposed, and the specific network structure design is shown in Fig 4.

On the backbone network based on VGG16, according to the characteristics of different types of disease radar data with different morphological distributions, the RFB module with wider sensing field is designed in the Conv3 feature layer and Conv7 feature layer to expand the network width so as to extract more detailed information about the disease from the original radar data; based on the design of RFB1, the fusion of RFB1, Conv4, Conv6 and Cbam modules are added to the three shallow feature layers. three shallow feature layers, the subtle features among different types of diseases can be recognized under strongly interfered radar data; consider adding the Cbam module on the two layers of Conv9 and Conv10, so that the model’s difference in the weight distribution for important information is more obvious; finally, based on the six network feature layer designs on the backbone network (38 × 38), (19 × 19), (10 × 10), (5 × 5), (3 × 3), and (1 × 1) a priori frames with different scale sizes are designed according to the six network feature layers on the backbone network, and the conforming a priori frames are screened by the method of non-maximum value suppression, which ultimately realizes the category prediction and accurate localization of the diverse feature diseases.

### Wide sensory field RFB module

Since the VGG16 backbone network adopts a direct-connection structure, as the backbone network of this model has the advantages of fewer network parameters, simple structure, and good classification performance, but it is ineffective in distinguishing the disease features from the background in the face of the underground diseases with diverse morphological features in the strong noise environment. The traditional RFB is a module based on the Inception [16] structure, which utilizes the convolution of different expansion rates to expand the sensory field of the feature network, and can extract the detailed features of the target.

**Fig 5:**
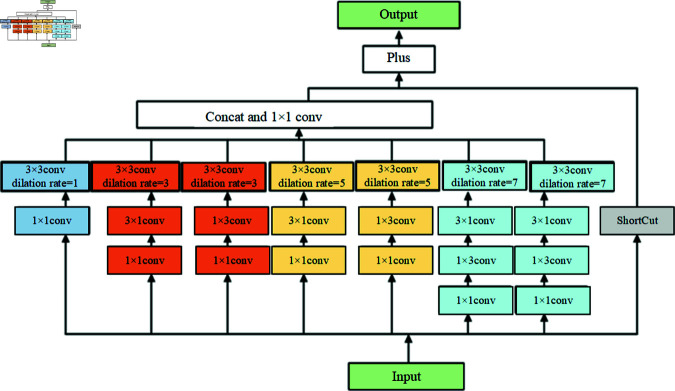
Wide feeling field RFB module.

Inspired by the RFB module, on the backbone network based on VGG16, this chapter designs a wider sensory field RFB structure. The specific structure is shown in Fig 5. The width of the network is enlarged by the convolution of 7 branches, and 2 groups of 3 × 1 and 1 × 3 convolutions are designed to enhance the depth of the network while reducing the amount of computation; 4 groups of expansion convolutions with expansion coefficients of 1, 3, 5, and 7 are designed to increase the receptive field. Finally, the feature map after the expansion convolution is fused with the 1 × 1 convolution through Concat, and the nonlinear convolutional layers are added by ShortCut to improve the information propagation efficiency, and all the feature results are fused by the addition operation.

#### RFB1 module design.

The Conv3 layer located in the backbone network of VGG16 has the characteristics of high resolution and small sensory field, and the RFB module is used under this feature layer can on the one hand, capture the detailed information such as texture and edges of the underground diseases well and learn the features of different types of underground diseases; on the other hand, due to the increase in the width of the model in the shallow network, when the network propagates backwards, it can reduce the loss of information in the original input image, which reduces the loss of features for detecting medium and large targets, and improves the detection of cracks, sparsity, and settlement type of disease.

#### RFB2 module design.

The 19 × 19 receptive field size of the Conv7 layer located in the VGG16 backbone network satisfies the network’s ability to extract the distinctive underground diseases. Since the size of the disease is distributed in the vicinity of the (19,19) feature layer with high probability, the introduction of the RFB module into this layer can make the network pay more attention to the center of the receptive field, which not only reduces the total number of parameters of the network model, but also improves the detection accuracy of the disease in a targeted way to prevent leakage of the detection.

### Scale feature fusion module (CONV4_FUS)

**Fig 6:**
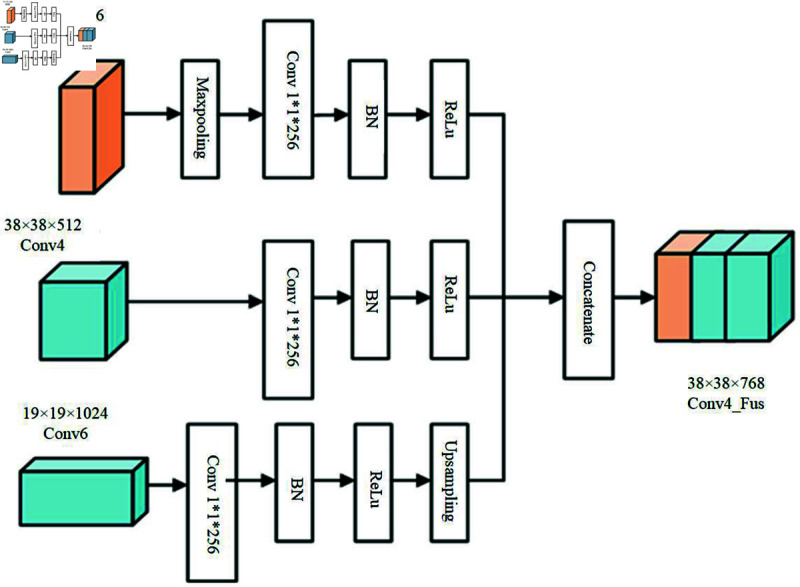
Conv4_Fus scale fusion module.

By designing a wider RFB module to optimize the network backbone of the model, the noise around the disease can be mitigated to a certain extent, but due to the ambiguity of the radar data features of different types of underground diseases, it is difficult to differentiate, and the network has a poor ability to capture the subtle differences between different types of diseases, especially for the cracks and dehiscence type of diseases, there is a low detection accuracy or even undetectable phenomenon. To address this problem, we consider mining the feature extraction capability of the shallow feature Conv4_3 layer, making full use of the high-resolution information of RFB1 and the rich semantic information of the Conv6 layer, fusing different sensory field features, and extracting highly recognizable detailed texture features of the diseases [17], to improve the model’s recognition accuracy of different types of diseases. Its specific network design structure is shown in Fig 6.

Conv4_Fus is a feature layer with a size of 38 × 38, formed by fusing RFB1, Conv4 and Conv6. Since the RFB1 module has a higher resolution than the Conv4 module and contains more detailed features of the disease, the RFB1 module is downsampled using the maximum pooling operation, setting the convolution kernel size to 2 × 2 and the step size to 2 to make the size uniform to 38 × 38.

Similarly, in order to unify the scale, the up-sampling operation is performed on the Conv6 module, and the convolution kernel size used is 2 × 2 with a step size of 2. Introducing 1 × 1 convolution on RFB1, Conv4, and Conv6 modules before fusion can reduce the number of input disease feature mappings and improve the computational efficiency. Due to the gap in the data dimension distribution of different feature layers, direct fusion will be ineffective in detecting diseases, and this chapter considers adding BN (Batch Normalization) layer on each feature layer for normalization before fusion. By designing this scale fusion module can enable the prediction network to take into account different disease details texture features and enhance the generalization ability of the model.

### Space channel attention mechanisms

Accurate extraction of deflated disease features is a prerequisite for improving the accuracy of detecting deflated disease. However, the convolutional neural network treats each channel and position of each feature map as equally important when performing feature extraction in, which is the same and lacks rationality in weight setting.The processing mechanism of the Cbam module [18] is similar to that of the human visual system, which views the field of view focusing the attention on the important and useful information and ignoring the unimportant information as a way of improving the classification accuracy.

**Fig 7:**
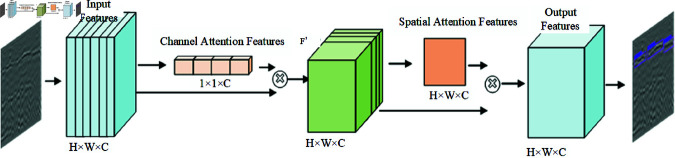
Cbam’s characteristic structure diagram.

Cbam module [19] is combined by channel attention module and spatial attention module, as shown in Fig 7 below, the input feature layer enters into the channel attention module first, and then enters into the spatial attention module after obtaining the feature maps with different weights of the channel, and the important features on the obtained output feature layer will be amplified and learned by the network. And located in the 5 × 5, 3 × 3 size of the feature layer for the important information on the weight distribution difference is more obvious, we consider adding Cbam module on these two layers, to retain more deglutition disease image information, obtained a more complete attention map, a better grasp of the global information.

#### Channel attention mechanisms.

**Fig 8:**
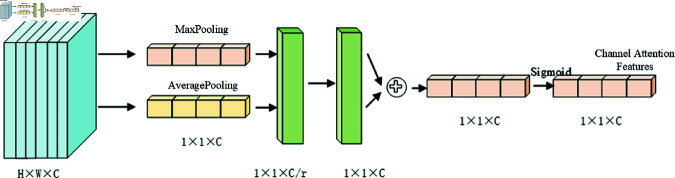
Structure diagram of Channel attention mechanism.

Fig 8 shows the typical structure of the channel attention module [21], which compresses the spatial dimensions of the feature mapping by using average pooling and maximum pooling for the input feature map; maximum pooling considers only the largest elements and ignores other elements in the region, which can retain more texture information of the subsurface disease image, and average pooling calculates the average value of all the elements in the pooling domain in order to retain more subsurface disease image background information. After the obtained results of average pooling and maximum pooling, the results are summed up using the shared fully connected layer, and finally the weights (between 0 and 1) of each channel of the input feature layer are obtained by the sigmoid function. After obtaining this weight, it is sufficient to multiply this weight by the original input feature layer.

#### Spatial attention mechanisms.

**Fig 9:**
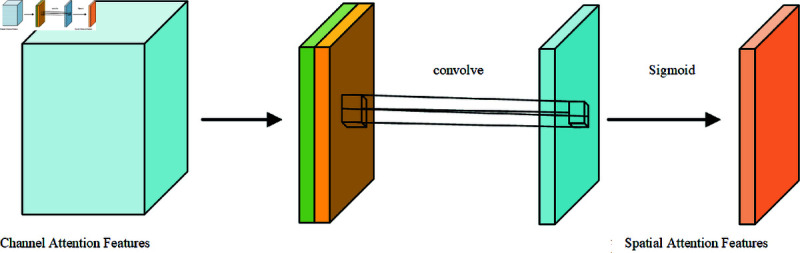
Structure diagram of spatial attention mechanism.

Fig 9 shows the typical structure of the spatial attention module [22]. The feature layers obtained through the channel attention mechanism described above are subjected to global maximum pooling and global average pooling on each feature plane, again obtaining two different feature layers, after which the two results are stacked one on top of the other, using a convolution with one channel at a time to adjust the number of channels. The sigmoid activation function is utilized to obtain the weights (between 0 and 1) of each feature point of the input feature layer. After obtaining this weight, it is sufficient to multiply this weight by the original input feature layer. Finally, the disease output feature information containing more comprehensive feature information is obtained.

### A priori boxed categorical regression design

The SF-Camb-SSD network uses a one-stage target detection method of classification regression on a priori frames for real-time detection of subsurface diseases. Firstly, six different scales of a priori frames are designed according to the six resolution feature layers of Conv4_Fus, RFB2, Conv8, Conv9, Conv10, and Conv11 on the backbone network, and the settings of the a priori frames include scale, aspect ratio, and number, and the scale settings of the a priori frames satisfy the following equation:

$$S_{k}=S_{min}+\frac{S_{max}+S_{min}}{m-1}(k-1),k\in [1,m]$$ (1)

$$min\_size=s_{k}\times input\_size$$ (2)

$$max\_size=s_{k-1}\times input\_size$$ (3)

Among them, *S*_*min*_ = 0.2, *S*_*max*_ = 0.9, *m* = 5, *S*_*k*_ indicate the scale of a priori frame size relative to the input map, using *S*_*k*_ to get min_size and max_size, firstly, two square a priori frames with side lengths of min_size and $\sqrt{min\_size\times max\_size}$ were generated, and then according to the distribution of the scale of the disease on the a priori frame aspect ratio and the number of settings to generate the a priori frames of $\sqrt{aspect\_ratio}\;\times \;min\_size$ and $\frac{1}{\sqrt{aspect\_ratio}}\;\times \;min\_size$ with side lengths of and to match with different sizes of the feature maps, which can be used for different sizes of the detection of the underground diseases, and the area of the a priori frames of each feature map is. The specific settings of the scale and the number of a priori frames are shown in Table 1. The area of each a priori box in each feature map is $area=[min\_size^{2},max\_size^{2}]$. The specific settings of the scale, aspect ratio and number of a priori boxes are shown in Table 1.

**Table 1:** Table of the number of underground diseases in four types of airports.

feature layer	size	number	area	aspect ratio
Conv4_Fus	38 × 38	6	[30,60]	2
RFB2	19 × 19	6	[60,111]	2,3
Conv8	10 × 10	6	[111,162]	2,3
Conv9	5 × 5	8	[162,213]	2,3,4
Conv10	3 × 3	4	[213,264]	3,4
Conv11	1 × 1	4	[264,315]	2

## Discussion

### Experimental environment

The hardware environment used for the experiments is: i7-8700 CPU with 3.2GHz server frequency, 64G RAM, 11G NVIDIA GeForce GTX 1080 Ti graphics card, and the operating system is Ubuntu 18.04. The TensorFlow deep learning framework is used for the training and testing work.

### Data sets

The experimental data in this paper are collected by ground-penetrating radar from three domestic airports with real data, probing the underground depth of 1.53 m, and the total collection area of $21083\;{\text{m}}^{2}$, and the collected radar map of underground diseases is cropped to a single-channel gray map of 448 × 448 pixels. The radar map was manually labeled based on expert experience, and finally the radar map was made into the corresponding PASCAL VOC [20] dataset format and named as AUD (Airport Underground Disease) dataset. In this chapter, 16040 radar maps containing four types of underground diseases, namely, cracks, dehollowing, settlement, and sparsity, were selected from the AUD dataset, and the number of each type of disease for the experiments in this chapter is shown in Table 2:

**Table 2:** Six kinds of scale a priori frame parameter setting table.

aspect ratio	DT-CRACK	DT-GAP	DT-LACUNAS	DT-SUBSIDENCE
Number of diseases	2120	55360	9430	5300

**Fig 10:**
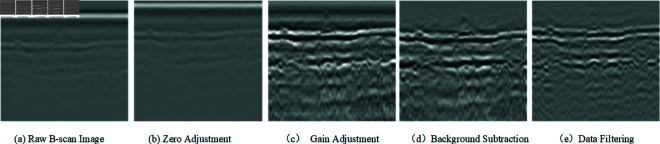
Pre-processing process of radar diagram.

In order for the model to better learn the morphological features of the disease, the radar map is preprocessed with traditional methods such as adjusting the zero point, adjusting the gain, background elimination, data filtering and other pre-processing operations at before the model training, which is used to filter out the clutter and eliminate the noise. The specific processing is shown in Fig 10:

### Network training parameter settings

During the 240 epochs of model training, the pre-trained model parameters were frozen for the first 140 epochs, allowing fine-tuning of the prediction layers by training the last six layers. In the subsequent 100 epochs, all model parameters were unfrozen for comprehensive training. The decision to utilize pre-trained weights was driven by several factors. Firstly, pre-trained weights provide a robust initialization for the EFA-SSD model, accelerating convergence during training. Secondly, they mitigate overfitting, which is particularly critical when handling limited radar images of airport underground defects. Finally, pretrained weights enable the transfer of general image features learned from the pretraining dataset to our radar-based defect detection task, thereby enhancing model performance. When the network is trained for 2 epochs, the loss value does not decrease compared to the loss value trained in the previous epoch, and the learning rate becomes 1/2 of the original.After completing 240 epochs of training, when the loss value does not decrease compared to the loss value trained in the previous epoch after training for 6 epochs, the network stops the training, and is considered to have reached the optimal value at this time. In this chapter, the training Batch Size is set to 16, the momentum size is set to 0.9, the weight decay is set to 1/2 of the previous one, and the learning rate is initially $5\times 10^{-3}$ reduced to $1\times 10^{-4}$ at the 141st epoch. In the training phase, this model uses the data enhancement of random cropping, horizontal flipping, and contrast transformation to expand the dataset randomly, and the resolution of the disease images is unified to 300 × 300 for training and testing.

### Comparative experiments

In order to validate the effectiveness of the proposed SF-SSD algorithm in detecting underground diseases in airports, the EFA-SSD was compared and experimented with five mainstream target detection algorithms, including SSD, Yolov8, Faster R-CNN, RFBNet, and RetinaNet algorithms.

Comparison experiments of various algorithms were conducted on the AUD dataset with an 8:2 ratio of training set to test set, an IoU of 0.5, and a confidence threshold of 0.5. Table 3 shows the detection results of various algorithms on various types of diseases, and the results show that the proposed EFA-SSD algorithm generally outperforms the SSD algorithm in terms of precision and recall of disease detection, and the overall mAP is improved by 26.6%.

**Table 3:** Compare experimental results.

IoU=0.5	DT-CRACK(%)			DT-GAP(%)			DT-LACUNAS(%)			DT-SUBSIDENCE(%)			mAP(%)	FPS
	Prec	Rec	F1	Prec	Rec	F1	Prec	Rec	F1	Prec	Rec	F1		
SSD	100	2.2	4.3	82.1	29.4	43.0	92.6	84.9	89.0	93.7	72.5	82.0	54.6	58.1
Yolov8	61.6	62.3	54.8	63.4	44.4	58.8	87.5	92.9	90.3	77.3	84.4	81.0	77.5	**63.2**
Faster R-CNN	21.3	80.0	34.0	34.2	89.6	49.0	77.0	95.3	65.0	40.7	90.2	56.0	73.1	7.5
RFBNet	89.3	30.5	45.0	88.6	44.6	59.0	90.0	94.2	**91.0**	96.1	77.5	86.0	79.7	53.4
RetinaNet	50.0	2.2	4.2	90.5	13.2	23.0	98.2	28.0	44.0	68.0	16.7	27.0	48.4	6.6
EFA-SSD	74.3	57.8	**65.0**	89.5	68.3	**77.0**	93.4	88.1	**91.0**	98.9	86.3	**92.0**	**81.2**	53.8

### Experimental results and analysis

Similarly, compared to other classical algorithms, the EFA-SSD algorithm exhibits significant precision and recall enhancement in recognizing diseases with varying morphology, such as cracks and dehulling, validating the effectiveness of its shallow scale fusion module. Ultimately, EFA-SSD achieves F1 values of 65%, 77%, 91%, and 92% for the four types of diseases, respectively, outperforming SSD, Yolov3, Faster R-CNN, RFBNet, and RetinaNet algorithms. Frames Per Second (FPS) is a crucial performance evaluation metric in object detection tasks, particularly in applications requiring real-time processing. This experiment also compares the FPS values of various models. The results show that the EFA-SSD model achieves an FPS of 53.8, meaning it can process approximately 53.8 images per second. In comparison, Yolov8 reaches an FPS of 63.2, while SSD achieves 58.1. Although EFA-SSD does not have the highest FPS, its high mAP value and relatively fast processing speed make it an ideal choice for real-time underground defect detection tasks on airport runways. Moreover, it retains potential for deployment on edge devices.

**Fig 11:**
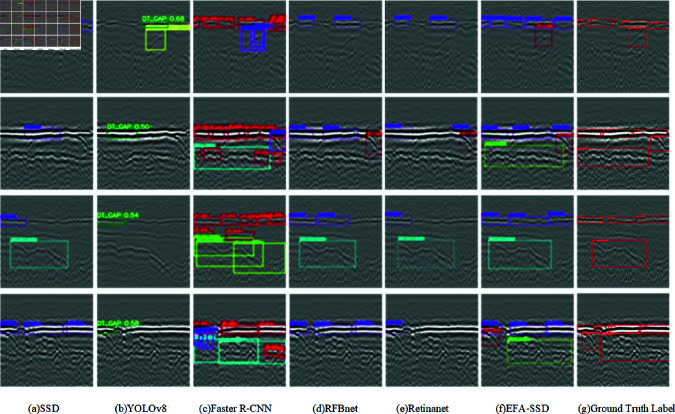
Comparison chart of visual inspection of underground diseases.

Fig 11 shows the visualized detection comparison between this algorithm and the other five detection algorithms. Fig 11(a) and 11(b) show that the SSD and YOLOv8 algorithms have obvious miss-detection in detecting boundary-ambiguous diseases such as subsidence and sparsity, and have low recall on dehollowing and cracking diseases.Faster R-CNN (Fig 11(c)) shows multiple redundant frames in the detection results, especially for dehollowing, which is basically the same disease with multiple frames, and it cannot realize the precise localization of the disease. RFBNet (Fig 11(d)) and RetinaNet (Fig 11(e)) also show the problems of missed detection and low confidence for various types of diseases, among which RetinaNet’s localization accuracy is worse and the missed detection rate is higher. In contrast, our EFA-SSD algorithm is able to accurately detect and provide a high level of confidence in the detection of both loose and settlement diseases with ambiguous boundaries, and dehollowing and cracking diseases with low recognition under complex background conditions.

**Fig 12:**
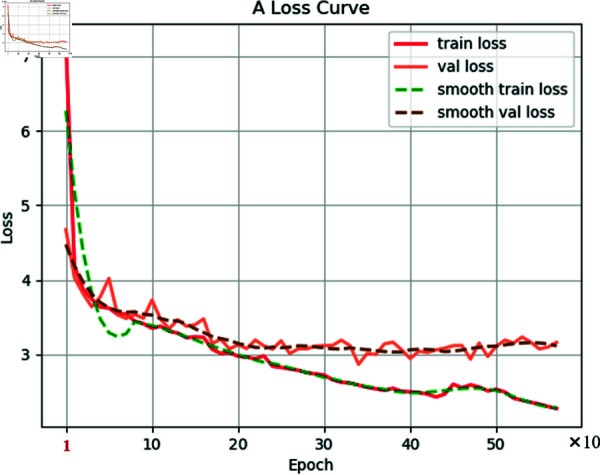
Loss curve.

Fig 12 illustrates the evolution of the Smooth L1 loss function during the training of the EFA-SSD model, including both training loss and validation loss along with their corresponding smoothed curves. It is evident from the figure that both training and validation losses exhibit a pronounced downward trend, indicating that the model effectively learns the features of airport runway underground defect data. In the initial training phase (0-100 epochs), the loss function decreases rapidly, suggesting that the model quickly adapts to the training data. As training progresses, the rate of decrease in the loss function gradually slows down and stabilizes in the later stages, indicating that the model has converged. However, after 442 epochs, the training loss exhibits a new downward trend, while the validation loss shows a slight upward trend, indicating that the model starts to overfit the training set beyond this point. Therefore, based on the loss function plot, it can be inferred that the model reaches its optimal performance at around 442 epochs.

### Ablation experiments

To verify the performance of each module in the EFA-SSD algorithm, ablation experiments were conducted. In the ablation experiments, except for the variable parameters, the rest of the network settings and training steps are kept the same. The ablation experiments were carried out sequentially in the above airport subsurface disease dataset to verify the effects of adding three modules independently, not adding three modules, and using three modules at the same time on the model, and the final experimental results (F1 values) were obtained as shown in Table 4 below:

**Table 4:** Compare experimental results.

Scale Fusion	RFB	Cbam	DT-CRACK(%)	DT-GAP(%)	DT-LACUNAS(%)	DT-SUBSIDENCE(%)	mAP(%)
			29.00	45.00	89.00	85.00	54.60
✓			63.00	50.00	87.00	86.00	70.50
	✓		51.00	60.00	88.00	86.00	74.25
		✓	44.00	45.00	90.00	87.00	66.50
✓	✓	✓	65.00	77.00	91.00	92.00	81.20

As shown in Table 4, when only the scale fusion module is added to the network significantly improves the F1 values of crack and dehollowing diseases, and the mAP is improved by about 16% compared to the baseline model, which indicates that the module effectively captures the shallow texture information of the disease radar data, and improves the detection accuracy of crack diseases. When only the RFB module is added, the F1 value of dehollowing disease is improved by 15%, which proves that the module expands the sensory field of the shallow network and extracts more disease features. The F1 values of both thinning and settling diseases were also improved when the Cbam module was added, which reduced the information loss of deeper features and suppressed the noise interference. The EFA-SSD algorithm using all three modules at the same time performs optimally in terms of F1 value and mAP value for all types of diseases.

To validate the effectiveness of the fusion module in the EFA-SSD algorithm, ablation experiments were conducted. By controlling other variables and maintaining a consistent experimental environment, we evaluated the impact of independently adding each of the two modules and simultaneously using all three modules on the model. The final experimental results (F1 values) are presented in Table 5 as follows:

**Table 5:** Ablation experiments on the multi-scale fusion module.

Conv4	Conv6	RFB1	DT-CRACK(%)	DT-GAP(%)	DT-LACUNAS(%)	DT-SUBSIDENCE(%)	mAP(%)
✓	✓		57.23	69.15	83.42	86.19	74.00
	✓	✓	62.58	74.29	89.01	90.58	79.12
✓		✓	59.85	72.41	86.29	88.92	76.87
✓	✓	✓	65.00	77.00	91.00	92.00	81.20

The experimental results show that when only the Conv4 and Conv6 modules are fused, the mAP value is 74%, indicating that the simple fusion of Conv4 and Conv6 fails to fully leverage the advantages of features from different layers. The fusion of Conv6 and RFB1 modules improves the mAP to 79.12%, validating the effectiveness of the RFB1 module in enhancing feature representation and improving detection accuracy. The fusion of Conv4 and RFB1 enhances the model’s ability to recognize different types of defects, but still falls short of achieving the best results. When all three modules(Conv4, Conv6, and RFB1) are fused, the mAP value of the experimental results reaches 81.20%, demonstrating that the fusion of these three modules maximizes their individual strengths and extracts more defect features from the original radar data.

## Conclusion

We study designed an improved EFA-SSD algorithm to address the challenges in the detection of subsurface diseases on airport roads. The EFA-SSD algorithm combines scale fusion and a wide sensory field RFB structure, which significantly improves the precision and recall of disease detection, and performs well especially in complex environments. The introduction of a spatial channel attention mechanism enhances the feature extraction capability, enabling the model to better capture the subtle features. The experimental results show that our algorithm effectively solves the problem of serious interference of morphological features of different types of diseases and low target recognition under strong noise background, which is of great significance for the detection of underground diseases in real airports.
